# Identification of mosaic and segmental aneuploidies by next-generation sequencing in preimplantation genetic screening can improve clinical outcomes compared to array-comparative genomic hybridization

**DOI:** 10.1186/s13039-017-0315-7

**Published:** 2017-04-26

**Authors:** Hsing-Hua Lai, Tzu-Hsuan Chuang, Lin-Kin Wong, Meng-Ju Lee, Chia-Lin Hsieh, Huai-Lin Wang, Shee-Uan Chen

**Affiliations:** 1 Stork Fertility Center, Stork Ladies Clinic, Hsinchu, Taiwan; 2 Department of Obstetrics and Gynecology, National Taiwan University Hospital and College of Medicine, Taipei, Taiwan

**Keywords:** Preimplantation genetic screening/chromosome mosaicism/segmental aneuploidy/next-generation sequencing

## Abstract

**Background:**

Chromosomal mosaicism is observed as the presence of both euploid and aneuploid cells in a particular blastocyst. Recent studies have reported that the implantation rate of mosaic embryo transfer is remarkably lower than the euploid embryos. The superior capability of next-generation sequencing (NGS) to detect chromosomal mosaicism in preimplantation genetic screening (PGS) remains controversial, and several data displayed similar implantation and pregnancy rates using NGS or array comparative genomic hybridization (aCGH).

**Results:**

In this study, the main inconsistency of aneuploidy detection and clinical performance between the NGS and aCGH were assessed. The phase I consisted of a parallel comparison in 182 blastocysts from 45 selected PGS patients for both the NGS and aCGH platforms. The phase II retrospectively compared the clinical outcomes of 90 patients with NGS-screened euploid embryo transfer to that of 129 patients with aCGH-screened euploid embryo transfer. The parallel comparison showed that the inconsistency of embryo euploidy was 11.8% (*p* = 0.01). Chromosomal mosaicism (10.7% with NGS *vs.* 3.9% with aCGH) and segmental aneuploidy (10.7% with NGS *vs.* 6.7% with aCGH) contributed to the discrepancy mainly. The chromosomally mosaic embryos (20%–50% of aneuploidy) and several embryos with segmental aneuploidy (≥10 Mbp) were hard to distinguish using the aCGH platform, but could be clearly identified using the NGS platform. After the first euploid embryo cryotransfer, the β-HCG(+) rate and implantation rate significantly increased in the PGS/NGS patients (HCG[+] rate: 73.3% in PGS/NGS *vs.* 60.5% in PGS/aCGH, *p* = 0.048; implantation rate: 53.2% in PGS/NGS *vs.* 45.0% in PGS/aCGH, *p* = 0.043). The clinical and ongoing pregnancy rates appeared higher in the NGS group, but did not reached statistical significance.

**Conclusions:**

The results demonstrated that the NGS platform can identify embryos with chromosomal mosaicism and segmental aneuploidy more precisely than the aCGH platform, and the following clinical performance of NGS was more favorable.

## Background

During the early stage of embryo development, chromosomal abnormalities (aneuploidies) often lead to growth arrest, repeated implantation failure, or recurrent miscarriage [1–3]. Embryonic aneuploidy is one of the main factors affecting the success rates of in vitro fertilization (IVF), and it originates from either a meiotic or a post-zygotic error. Increased maternal age is the primary cause of most meiotic errors, which occur during oogenesis. Other contributions to embryonic aneuploidy are the errors that arise after fertilization; even embryos produced by fertile couples could be aneuploid due to random mitotic flaws. Nonetheless, the aneuploid rate is comparatively higher for the couples undergoing IVF than for the fertile couples. In IVF-produced embryos, post-zygotic chromosomal abnormalities happen more frequently than the meiotic errors [4].

The presence of two or more distinct cell lines that exist in the same embryo is known as mosaicism, which is mainly caused by mitotic errors [5, 6]. Improper segregation of chromosomes in the first three cleavage divisions is reported to have the highest tendency toward mosaicism [7]. The three main mechanisms by which chromosomal mosaicism occurs, leading to a gain or loss of chromosomes, include anaphase lagging [8, 9], endoreplication [10], and non-disjunction [11]. Compared with the cleavage-stage embryos created by IVF, chromosomal mosaicism persists but occurs to a lesser extent in the blastocyst stage [12], in which these mosaic blastocysts have varying degrees of mosaicism. According to Fragouli et al [13], most mosaic blastocysts have different abnormalities in every cell (chaotic), but around 10% of these mosaics contain both diploid and aneuploid cells.

Because the embryonic aneuploidy affects IVF outcomes, selection of embryos without chromosomal aneuploidy for transfer using reliable comprehensive chromosome screening (CCS) techniques is a critical issue in the field of preimplantation genetic screening (PGS) [14–16]. Advances in CCS/PGS led to the development of array-comparative genomic hybridization (aCGH), which has become a widely-used method to analyze the whole chromosome copy numbers [17–19]. The aCGH platform is employed to analyze the chromosomal aneuploidy in metaphase II (MII) oocytes, cleavage stage embryos, and blastocysts [20, 21], and it indeed improved the clinical outcomes of IVF patients [22]. However, some limitations of the worldwide aCGH platform restrict it from detecting low-rate aneuploidy (chromosomal mosaicism) and segmental aneuploidy in biopsied trophectoderm (TE) samples. The narrow dynamic range for interpretation, human errors during the manipulation process, or inconclusive results caused by biological effects may introduce natural or artificial bias, which hampers the detection of chromosomal mosaicism and segmental aneuploidy in the embryos (A technical guide to aneuploidy calling with 24sure V3, Illumina).

Recently, next-generation sequencing (NGS) was introduced in the reproductive medicine as a new methodology for CCS [23–25]. Compared to the aCGH platform, NGS is a sequencing-based analysis technique and thus has higher resolution (about 2 mega base pairs, Mbp) and broader dynamic range (copy-number scale) for interpretation [23, 24]. Intriguingly, the consistency reported by several studies displayed the same efficiency in comprehensive aneuploidy screening between the aCGH and NGS platforms [26–28], and the author also observed that the identification of chromosomal mosaicism by NGS could be more sensitive than aCGH [27]. Still, there is no well-designed study to compare these two CCS platforms in a double-blind fashion by two independent laboratories.

According to the recent report released by the European Society of Human Reproduction and Embryology (ESHRE), the implantation rate of mosaic embryo transfer is significantly lower than that of euploid embryos [29]. Although a few mosaic embryos have the capacity of implantation or even live birth, the success rate is low [30]. Therefore, we performed a parallel comparison between the aCGH and NGS platforms to investigate the main cause of inconsistent results, and then compared the clinical outcomes of euploid embryo transfer by PGS/NGS with those by PGS/aCGH. We hypothesized that the main contribution to the inconsistency of the two CCS platforms was mosaic embryo identification, and clinical outcomes could be improved after excluding mosaic embryo transfer by screening with NGS.

## Methods

### Study design

This study was a retrospective analysis involving women with an indication for PGS in their IVF programs between 2014 and 2015. The study was approved by the appropriate ethics reviewing committee of National Taiwan University Hospital (Institutional Review Board Number: 201510127RIND). All patients were from the outpatient department of a fertility center (Hsinchu, Taiwan), and counseled by fertility specialists regarding the PGS. Consistency between the NGS and well-validated aCGH was assessed in the selected samples of 45 patients (182 blastocysts). In phase I, the embryos in the parallel comparison between NGS and aCGH were processed for TE biopsy, which was followed by whole genome amplification (WGA) and whole chromosome screening. The results of two platforms were compared for consistency. In phase II, the clinical outcomes of 90 women with PGS/NGS screened embryo transfers in 2015 were compared to those with 129 PGS/aCGH screened embryo transfers in 2014.

### Study objects

In the parallel comparison, 45 women were 21–42 years of age (mean age: 35.5 years). The patients with severe male infertile factors, advanced maternal age (≥36 years), history of repeated implantation failure, or using donated oocytes for single embryo transfer, and planned to undergo PGS in their IVF cycles were included. The patients underwent a complete consultation involving the possible advantages, previously reported success rates, and risks of misdiagnosis of the aCGH and NGS. In the comparison of clinical outcomes, 90 patients with PGS/NGS screened embryo transfers in 2015 and 129 patients with PGS/aCGH screened embryo transfers in 2014 displayed similar demographics. Written informed consents were obtained from all the couples included in the study.

### IVF and embryo biopsy procedure

The patients were treated with individualized stimulation protocols [31], following with oocyte retrieval operation. The MII oocytes were fertilized and cultured at 37 °C, 6.0% CO_2_, 5.0% O_2_. Embryos were cultured in groups, and droplets of one-step human embryo culture media (Global, LifeGlobal, USA) under mineral oil were used. A hole in the zona pellucida was made by laser (Saturn 5, Research Instrument, UK) in most 4-day-old embryos to assist embryo hatching. On day 5, the blastocysts with inner-cell mass (ICM) grading ≥ B [32] and a distinct cellular TE were biopsied. The other blastocysts that did not meet the criteria on day 5 were evaluated again on day 6 and day 7 for the possibility of biopsy.

In the biopsy procedure, around 5–10 TE cells were aspirated using a biopsy pipette (Origio, Måløv, Denmark) and then removed by shearing force between the biopsy pipette and holding pipette. The procedure was performed with micromanipulation equipment in droplets of PGD Biopsy medium (Global, LifeGlobal, USA). The biopsied TE cells were washed in sterile 1x phosphate-buffered saline (PBS) solution (Cell Signaling Technology, Cell Signaling Technologies, USA) containing 1% polyvinylpyrrolidone (PVP) solution (Sigma, Sigma-Aldrich, USA) twice. The washed cells were gently expelled into a 0.2-ml PCR tube with 2.5 μl of PBS/PVP to the following amplification, and then the blastocysts were vitrified. Details of the procedure can be found in Chang et al., 2013 [33].

### Whole-genome amplification, and DNA quantification

The biopsied cells were lysed, and the released genomic DNA was fragmented. The fragmented DNAs were amplified according to the manufacturer’s procedures for the Sureplex WGA system (Sureplex, Illumina, USA). Succinctly, the biopsied cells were lysed by Sureplex cell extraction buffer and cell extraction master mix. Then they were incubated at 75 °C for 10 min followed by 95 °C for 4 min. The released genomic DNAs were randomly fragmented in Sureplex pre-amplification cocktail and incubated according to the following program: 1 cycle of 95 °C for 2 min, and 12 cycles of 95 °C for 15 s, 15 °C for 50 s, 25 °C for 40 s, 35 °C for 30 s, 65 °C for 40 s, 75 °C for 40 s, and holding at 4 °C. Finally, Sureplex amplification cocktail was added and the final program was as: 14 cycles of 95 °C for 15 s, 65 °C for 1 min, 75 °C for 1 min, and holding at 4 °C. The dsDNA High-Sensitivity (HS) Assay Kit (Qubit®, Life Technologies, USA) was used to quantify the concentration of amplified DNAs. After biopsy, the sample was treated with WGA. The amplification products from the 45 patients of the first phase were divided into two portions for the following aCGH and NGS analyses.

### aCGH analysis

The amplified WGA products were assessed by aCGH testing with 24sure V3 microarray (Illumina, Inc.) at the Genesis Genetic Asia Laboratory (Taiwan, Taipei). The products and reference DNAs were labeled with Cy3 and Cy5 fluorophores using random primers for 2–4 h. Then the labeling mixes were combined and co-precipitated with COT Human DNA in preparation for hybridization. Labeled DNAs were re-suspended in a dexsulphate hybridization buffer and hybridized onto the 24sure chip for 12 h. Thereafter, the chips were washed and dried. A laser scanner was used to read the resulting images, and BlueFuse Multi Software (Illumina, Inc.) was used to analyze the scan data. More details of aCGH testing procedure can be found in Huang et al., 2013 [34]. Once a specific amplification was observed, autosomal profiles were analyzed for gain or loss whole chromosomal ratios, using a 3 x SD assessment, greater than ± 0.3log2 ratio call, or both. For hybridization quality control, female samples hybridized with a male reference (sex mismatch) had to show a consistent gain of chromosome X and a consistent loss of chromosome Y [35].

### NGS analysis

The same WGA product of each sample was processed to prepare DNA libraries at the PGS laboratory of Stork Fertility Center (Hsinchu, Taiwan), by following the manufacturer’s guidelines for VeriSeq PGS (Illumina, Inc). The diluted DNA (0.2 ng/μl, 1 ng total) was tagged and fragmented (“tagmented”) using the Nextera XT transposome (Amplicon Tagmentation Mixture and Tagmentation DNA Buffer) through a limited-cycle PCR reaction. Then the index sequences were added to the samples to enable dual-indexed sequencing (2 x 36 bp). The tagmented DNAs with added indexes were amplified using the Nextera PCR Master Mix (NPM) through a PCR program: 1 cycle of 72 °C for 3 min, and 12 cycles of 95 °C for 10 s, 55 °C for 30 s, 72 °C for 30 s, 1 cycle at 72 °C for 5 min, and holding at 4 °C.

The PCR products were cleaned using the AMPure XP beads (A63881, Beckam Coulter, USA), providing a size selection from the population. After processing, the purified libraries were washed with 80% ethanol solution, then they were eluted by Nextera XT Resuspension Buffer. The purified DNA libraries were then normalized to equalize the quantity of each sample in the final pooling using the Library Normalization Additive and beads. Then, the normalized samples with equal volumes were pooled, denatured, and then sequenced. The Miseq Reagent Kit v.3 (Illumina, Inc) was used on a Miseq System (Illumina, Inc). The generated bioinformatics data was also analyzed by BlueFuse Multi Software (Illumina, Inc). Embryos were identified if they displayed a median chromosomal copy number deviated from the default copy number, and a possible trisomy or monosomy of autosomal chromosomes was seen as a copy number > 2 or < 2, respectively. Details of preparation procedures and the determination principles of automated copy number for each chromosome on BlueFuse Multi Software (Illumina, Inc.) were described in Fiorentino et al., 2014 [26].

### Mosaicism identification

The detection sensitivity to chromosomal mosaicism on both CCS platforms were determined by a mixing experiment (Fiorentino, 2014 ESHRE abstract) [36]. The sample with whole chromosome loss/gain (100% of monosomy/trisomy) were serial diluted with euploid sample (50%, 40%, 30%, 20%, 10% of aneuploidy), and then were tested on the two CCS platforms. With aCGH system, the X-separation was used to distinguish mosaic embryos [28]. If the absolute log_2_ value of an aneuploidy was higher than half of the X-separation, this aneuploidy was determined as “pure,” since the all tested cells in the fraction were like with this aneuploidy. In contrast, if the absolute log_2_ value of an aneuploidy was lower than half of the X-separation, it would be determined as mosaic, since this fraction could contain both euploid and aneuploid cells. With the NGS system, samples with ≥ 50% aneuploidy (>2.5 or < 1.5 copy number) could be distinguished clearly, and samples with aneuploidy between 20% and 50% (2.0 ± 0.2–0.5 copy number) could be observed only under the finest background (overall noise < 0.20). According to Greco et al., 2015 [30], the live births has been reported only in the transfers of mosaic embryo with under 50% of aneuploidy, and thus the embryos with aneuploid percentage between 20% and 50% was classified as mosaic (low-rate aneuploid). The embryos with aneuploid percentage under 20% was classified as euploid, and with aneuploid percentage over 50% as aneuploid.

### Clinical outcomes and definition

The serum β-hCG was measured 2 weeks after cryotransfer of one or two euploid blastocysts, and if positive, transabdominal ultrasonography was performed at 7 weeks gestation. Once the gestational sac and fetal heartbeat were detected, the patient was considered to have achieved a clinical pregnancy. In contrast, the absence of an identifiable gestational sac was defined as “chemical pregnancy.” After 16 weeks of gestation, the patient was included in the ongoing pregnancy group. The number of fetal heartbeat-positive pregnancies per transferred blastocyst was defined as the implantation rate (shown as a percentage). The miscarriage rate was defined as the number of 16-week pregnancies lost per cryotransfer (shown as a percentage).

### Statistical analysis

The count data were presented as percentages, and the continuous data as averages with standard deviations (SDs). Multiple parameters compared between the groups were analyzed using the Student *t* test or Manny-Whitney *U* test, depending on the population distribution. Comparisons of percentage distribution between the groups were analyzed by Chi-square test. Significant differences were defined as two-sided *p*-value <0.05. All the analyses were generated using scientific GraphPad software (Prism, GraphPad Software, USA).

## Results

### Patient profile

Forty-five patients (mean age = 35.5 years, range 21–42) selected from the study cohort undergoing PGS were involved in this parallel comparison of NGS and aCGH (Table 1). The causes of infertility included severe male factor (5/45, 11.1%), advanced maternal age (≥36 year, 20/45, 44.4%), repeated implantation failure (14/45, 31.1%), donated-oocyte recipients (6/45, 13.3%). Among all patients, 182 blastocysts were harvested and biopsied. Excluding the samples that failed to amplify, 178 embryos were screened by the NGS and aCGH.

**Table 1:** Patient profile of parallel comparison

Patient number	45
Mean female age (years)	35.5 (21–42)
Baseline AMH(ng/mL)^a^	4.0 ± 3.7
Antral follicle count^a^	8.9 ± 4.4
Indications^b^	
Severe male factor	5 (11.1%)
Advanced female age (≧36 years)	20 (44.4%)
Repeated implantation failure	14 (31.1%)
Donated-oocyte recipient for single embryo transfer	6 (13.3%)
Number of embryos biopsied (≧ grade BC)	182
Number of embryos with WGA failure	4
Number of embryos screened by PGS	178

*AMH* anti-Mullerian hormone, *WGA* whole genome amplification, *PGS* preimplantation genetic screening

^a^Data are presented as mean ± SD

^b^Data are presented as the number of the class (percentage of the class)

### Parallel comparison

Table 2 showed the results of double-blind interpretation of NGS and aCGH screening in 178 blastocysts obtained from the 45 patients. In the consistency assessment of embryo ploidy, aneuploid (51.1% with NGS *vs.* 46.1% with aCGH) and mosaic embryos (10.7% with NGS *vs.* 3.9% with aCGH) identified were both higher with NGS than aCGH. The percentage of embryos diagnosed as abnormal (aneuploid and mosaic) by NGS was 61.8%, while the percentage for aCGH was 50.0%. The overall inconsistency of embryo euploidy between the two CCS platforms was 11.8% (21/178, *p* = 0.01).

**Table 2:** Parallel comparisons between NGS and aCGH

	NGS	aCGH	*P*-value
Patient number	45		
No. of embryo screened	178		
Euploid (%)	68 (38.2%)	89 (50.0%)	0.01*
Aneuploid (%)	91 (51.1%)	82 (46.1%)	
Mosaic (%)	19 (10.7%)	7 (3.9%)	
Inconsistency of embryo euploidy^a^	21 (11.8%)		
Aneuploidy assessment			
No. of aneuploid embryo	91	82	
Complex aneuploidy (%)	26 (28.5%)	24 (30.5%)	0.78
Trisomy (%)	14 (15.4%)	13 (14.6%)	
Monosomy (%)	32 (35.2%)	33 (40.2%)	
Segmental aneuploidy (≥10 Mbp) (%)^b^	19 (20.9%)	12 (14.6%)	
Inconsistency of embryo aneuploidy^c^	9 (5.1%)		
Mosaicism assessment			
No. of mosaic embryo	19	7	
Whole chromosomal mosaicism	16 (84.2%)	5 (71.4%)	0.59
Segmental chromosomal mosaicism	3 (15.8%)	2 (28.6%)	
Inconsistency of chromosomal mosaicism^d^	12 (6.7%)		

*NGS* next-generation sequencing, *aCGH* array-comparative genomic hybridization

*P-values <0.05 are defined as statistically significant, and they are calculated by Chi-square analysis

^a^The number of euploid embryos were different on the two platforms

^b^The segmental aneuploidy was defined as the variation length reaching 10 Mbp by the both two laboratories

^c^The number of aneuploid embryos were different on the two platforms

^d^The detected chromosomal mosaicism were different on the two platforms

**Fig. 1:**
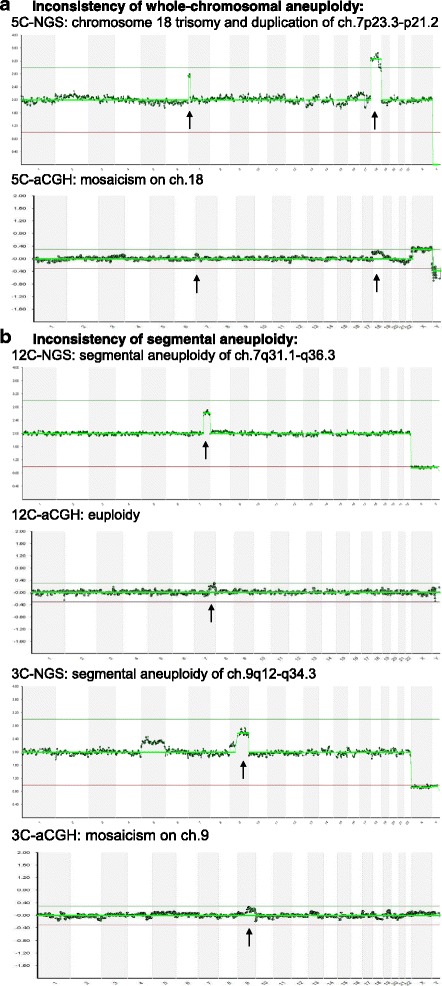
Examples of inconsistent aneuploidy between the two chromosome screening platforms generated by the same amplification products. NGS, next-generation sequencing; aCGH, array-comparative genomic hybridization. **a** Embryo 5C was identified as aneuploid with trisomy 18 and duplication of ch.7p23.3-p21.2 using the NGS platform, but the aneuploidy was suspected as mosaicism at ch.18 using the aCGH platform. **b** Embryo 12C was identified as aneuploid with duplication of ch.7 q31.1-q36.3 using the NGS platform (47 Mbp), but the aneuploidy was not obvious with the aCGH platform. Embryo 3C was identified as aneuploid with duplication of ch.9q12-q34.3 using the NGS platform (83 Mbp), but the aneuploidy was suspected as mosaicism with the aCGH platform

In the consistency assessment of aneuploid embryos, NGS detected 26 embryos with complex aneuploidy (≥3 variations), but 2 embryos in them were identified as merely monosomy and segmental aneuploidy, respectively, with the aCGH. An aneuploid embryo with both the ch.18 trisomy and duplication of ch.7p23.3-p21.2 on the NGS was identified as a mosaic on ch.18 by the aCGH (Fig. 1a). Eight aneuploid embryos with segmental variations (mean length = 30.8 Mbp) identified with the NGS were not detected on the aCGH (6 euploids and 2 suspected mosaics, Fig. 1b). Thus, the overall inconsistency of chromosomal aneuploidy between the two platforms was 5.1% (9/178, *p* = 0.78); the main discrepancy was the detection of segmental aneuploidy (10.7% with NGS, 19/178 *vs.* 6.7% with aCGH, 12/178).

**Fig. 2:**
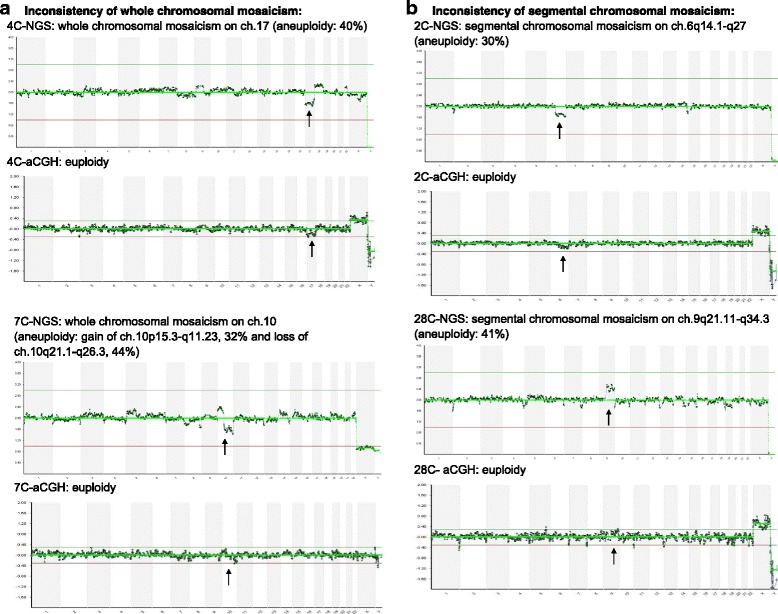
Examples of inconsistent mosaicism between the two chromosome screening platforms generated by the same amplification products. NGS, next-generation sequencing; aCGH, array-comparative genomic hybridization. **a** Embryo 4C was identified as mosaic with partial deletion of ch.17 (40% of aneuploidy) using the NGS platform, but the mosaicism was not obvious with the aCGH platform. Embryo 7C was identified as mosaic with both the partial duplication of ch.10p15.3-q11.23 (32% of aneuploidy) and partial deletion of ch.10q21.1-q26.3 (44% of the aneuploidy) using the NGS platform, but the mosaicism was not obvious with the aCGH platform. **b** Embryo 2C was identified as mosaic with partial deletion of ch.6q14.1-q27 using the NGS platform (30% of aneuploidy), but the segmental chromosomal mosaicism was not detected using the aCGH platform. Embryo 28C was identified as a mosaic with partial duplication of 9q21.11-q34.3 using the NGS platform (41% of aneuploidy), but the segmental chromosomal mosaicism was not detected using the aCGH platform

In the consistency assessment of chromosomal mosaicism in the mosaic embryos, 12 mosaic embryos with whole chromosomal mosaicism identified with the NGS (mean aneuploid percentage = 34.4%) were not found by the aCGH (12 euploids, Fig. 2a). Additionally, three mosaic embryos with segmental chromosomal mosaicism on NGS (mean aneuploid percentage = 33.3%; mean length = 73 Mbp) were not detected by the aCGH (3 euploids, Fig. 2b). Only four mosaic embryos with chromosomal mosaicism were identified by both the two screening platforms, and three mosaic embryos detected by aCGH were identified as aneuploid embryos by NGS (aneuploid percentage >50%). Therefore, the overall inconsistency of chromosomal mosaicism between the two platforms was 6.7% (12/178, *p* = 0.59), and mosaicism detection was the main cause of inconsistency between the two platforms (10.7% with NGS, 19/178 *vs.* 3.9% with aCGH, 7/178).

### Clinical outcomes

Table 3 displayed the reproductive outcomes of the 90 patients with PGS/NGS-diagnosed embryo transfer in 2015 and the 129 patients with PGS/aCGH-diagnosed embryo transfer in 2014. Of the initial PGS results, the beginning number of patients undergoing PGS was 135 in the NGS group and 202 in the aCGH group; the number of PGS-screening embryos was 472 in the NGS group and 827 in the aCGH group, respectively. The detection rate of mosaicism was significantly higher in the NGS group of 5.3% (25/472) than in the aCGH group of 1.7% (14/827) (*p* < 0.01), while the rate of segmental aneuploidy showed no difference between the two groups (10.2% [48/472] *vs.* 9.6% [79/827], *p* = 0.77). The patients with euploid embryos were enrolled in this study for transfer, and the patients with no euploid embryos were excluded. The percentage of exclusion was 32.6% (44/135) in the NGS group, greater than 20.8% (42/202) in the aCGH group (*p* = 0.02). Of the comparisons of clinical outcomes, no significant differences were observed in the baseline characteristics of patients undergoing transfer(s), including the female age, infertility indication, anti-Müllerian hormone (AMH), antral follicle count (AFC), and mean endometrial thickness before transfer. The β-HCG positive rate and implantation rate after the first cryotransfer were significantly higher in the NGS group than the aCGH group (HCG(+) rate: 73.3% *vs.* 60.5%, *p* = 0.048; implantation rate: 53.2% *vs.* 45.0%, *p* = 0.043). The clinical pregnancy rate and ongoing pregnancy rate appeared higher, and the miscarriage rate was lower in the NGS group than those in the aCGH group. However, the difference did not reach statistical significance. The average number of transferred embryos in both groups was 1.2 blastocysts.

**Table 3:** Clinical outcomes of patients undergoing NGS and aCGH

	NGS	aCGH	*P*-value
Number of patients undergoing PGS	135	202	—
Number of embryos diagnose by PGS^a^	472	827	—
Euploid	180 (38.1%)	364 (44.0%)	0.04*
Aneuploid	219 (46.4%)	370 (44.7%)	0.60
Mosaic	25 (5.3%)	14 (1.7%)	<0.01**
Segmental aneuploid	48 (10.2%)	79 (9.6%)	0.77
Number of patients with no euploid embryo to transfer^a^	44 (32.6%)	42 (20.8%)	0.02*
Aneuploid	23 (17.0%)	32 (15.8%)	0.77
Mosaic	6 (4.4%)	0 (0%)	—
Segmental aneuploid	15 (11.1%)	10 (5.0%)	0.05
Number of transferred patients^a^	90 (66.7%)	129 (63.9%)	—
Mean female age (years)^b^	37.5 ± 5.5 (27–53)	37.2 ± 4.5 (27–51)	0.68
Indications^a^			
Severe male factor	9 (10%)	25 (19%)	0.08
Advanced female age (≥36 years)	32 (36%)	42 (33%)	
Repeated implantation failure	32 (36%)	47 (36%)	
Oocyte –donation cycle for SET	17 (19%)	15 (12%)	
Baseline AMH (ng/mL)^b^	4.5 ± 3.7	4.9 ± 3.6	0.42
Antral follicle count^b^	11.0 ± 4.6	11.2 ± 4.7	0.82
Mean endometrial thickness (mm)^b^	9.3 ± 1.8	9.2 ± 1.4	0.72
Clinical outcomes after the first cryotransfer^a^			
HCG(+) pregnancy	66 (73%)	78 (60%)	0.048*
Implantation	64 (53%)	67 (45%)	0.043*
Clinical pregnancy	59 (66%)	73 (57%)	0.18
Ongoing pregnancy	51 (57%)	59 (46%)	0.11
Multiple pregnancy	3 (3%)	4 (3%)	1.00
Miscarriage	8 (9%)	14 (11%)	0.48
Average transferred embryo per cryotransfer^b^	1.2 ± 0.5	1.2 ± 0.4	0.11

*AMH* anti-Müllerian hormone, *NGS* next-generation sequencing, *aCGH* array-comparative genomic hybridization, *SET* single embryo transfer

*P-values <0.05 indicates statistical significance, and they are calculated by either the Mann-Whitney *U* test or Chi-square test depending on the population

^a^Data are presented as the number of the class (percentage of the class)

^b^Data are presented as mean ± SD

## Discussion

The present analysis is the first study to demonstrate that the PGS/NGS platform can identify embryos with chromosomal mosaicism and segmental aneuploidy more precisely than the PGS/aCGH platform. Furthermore, we showed that the clinical outcomes of patients with NGS-screened embryo transfer had significantly better results than those with aCGH-screened embryo transfer after excluding the transfers of mosaic and segmental aneuploid embryos.

The study included a parallel comparison and a clinical outcome comparison between the two CCS platforms. In the first parallel comparison, the amplified products of samples were analyzed with both the NGS and aCGH, which were produced by the same manufacture (Illumina, San Diego, USA). The analyses of raw data were conducted using the same software (BlueFuse Multi, Illumina, Inc.), and the final interpretations were determined by the two independent laboratories in a double-blind fashion. The inconsistency between the two CCS platforms was 11.8% (*p* = 0.01). This discrepancy was mainly due to the identification of mosaicism (10.7% with NGS *vs.* 3.9% with aCGH), including both whole chromosomal mosaicism (9.0% with NGS *vs.* 2.8% with aCGH), and segmental chromosomal mosaicism (1.7% with NGS *vs.* 1.1% with aCGH). The total percentage of mosaic embryos detected with NGS in the study (10.7%) was similar to that of Fragouli et al., 2011, around 10% [13]. The second cause of inconsistency was the detection of segmental aneuploidy (10.7% with NGS *vs.* 6.7% with aCGH). The reported incidences of segmental aneuploidy varied broadly between studies [28]. The total percentage of segmental-aneuploid embryos detected with NGS in this study was slightly higher than the past data, which the aCGH was used [37], around 6%–7%.

The robust detection power of NGS platform resulted in a more accurate screening of embryos with low-rate and segmental aneuploidies compared to aCGH, which reflected in the following comparison of clinical outcomes. Since the lower implantation rate and live births of mosaic embryo has been reported [29, 30], we excluded the patients with mosaic embryo transfer in the phase II comparison to assess the clinical performance in euploid embryos of two platforms. With similar patient demographics, the patients with NGS-screened embryo transfer achieved significantly higher HCG(+) rate (73.3% *vs.* 60.5%, *p* = 0.048) and implantation rate (53.2% *vs.* 45.0%, *p* = 0.043) than patients with aCGH-screened embryo transfer. Possibly due to the sample size, the clinical and ongoing pregnancy rates did not reach significant difference between two arms, and the NGS group displayed more favorable. We have concluded that NGS screened embryo transfer had significantly better results than aCGH after control the proportion of mosaic or segmental aneuploidy, which could influence the clinical outcome of IVF. The percentages of patients excluded for transfer due to mosaicism or segmental aneuploid in the NGS group appeared higher than those in the aCGH group, but no significance reached yet (mosaic: 4.4% *vs.* 0%; segmental aneuploid: 11.1% *vs.* 5.0%, *p* = 0.05). The results implied that the efficacy of single euploid embryo transfer may be effectively improved by the NGS screening due to the more rigorous identification of mosaic and segmental-aneuploid embryos, whose competency deserves further study to validate [25, 29, 30].

Accordingly, NGS possesses two advantages compared to aCGH for the identification of low-rate and segmental aneuploidies: a broader dynamic range for interpretation of low-rate aneuploidy (mosaicism) and increased chromosomal resolution of about 2 Mbp to detect segmental aneuploidy. Moreover, a library-based sample preparation could also decrease the background noise caused by artifacts, which may affect the final aneuploid results [26, 38]. Nonetheless, several data have reported that no significant differences were found in detection of mosaicism or segmental aneuploidy between the NGS and aCGH platforms in strict parallel comparisons [27, 28, 39]. In contrast, the present study showed that identification of both mosaicism and segmental aneuploidy differed significantly between the NGS and aCGH platforms when mosaicism was defined as an aneuploid rate between 20% and 50% (low-rate aneuploidy) [30], and segmental aneuploidy was defined as ≥ 10 Mbp [40]. Since both the demographics of patients and sample size of parallel comparison in our study were very similar to those of Yang et al. in 2015 [27], the opposite results could be due to the different definitions used for data interpretation.

Chromosomal mosaicism is common during preimplantation development. It was once reported as 65% and 70% during the early mitotic stage [41, 42], and as 10% during the blastocyst stage [4]. The chromosomal mosaicism was also observed in the prenatal tests of non-IVF people, including chorionic villus (about 1.78%) and amniotic fluid (about 0.46%) [43]. The propagation of abnormal cell line(s) in the mosaic embryos had negative effect in the pregnancy, and the undetected mosaicism and aneuploidies in the PGS/aCGH was recently reported as a cause to the first trimester pregnancy loss [44]. However, the IVF patients with single mosaic embryo transfer could still achieve live births according to Greco el al., 2015 [30], and thus the competency of mosaic embryo remained uncertain. Additionally, the incidence of confined placental mosaicism (CPM) would also be a bias to the accuracy of PGS, which the trophectoderm samples were used [12]. According to our preliminary data during 2015 and 2016, two out of ten patients with NGS-diagnosed mosaic embryo transfer had normal reports of amniocentesis (both karyotyping and array screening) and then achieved live births (data not shown).

Although excluding mosaic and segmental aneuploid embryo transfer based on NGS identification led to significantly higher HCG(+) rate and implantation rate in this study, some patients with advanced maternal age may not have any euploid embryo to transfer and must consider mosaic embryo transfer instead. Thus both complete consultations before mosaic embryo transfer and appropriate prenatal testing after mosaic embryo transfer are mandatory in these patients. For mosaic embryos, we should counsel the possibility of discarding a competent embryo versus transferring an embryo that may have a lower implantation potential and possible adverse obstetrical and neonatal outcomes [45]. In this study, six out of 44 patients only had mosaic embryos to consider transfer in the NGS group, and none in the aCGH group. After complete consultation, all of them choose to transfer the unscreened embryos rather than transferring the mosaics [46].

The study is also restricted by its retrospective nature. Certainly, it is not known whether mosaic or segmental-aneuploid embryo transfers were actually involved in the outcomes of PGS/aCGH group, and it is only assumed that the reduced clinical outcomes of patients with aCGH-screened embryo transfer were due to the methodological limitation of chromosomal mosaic detection and segmental aneuploidy identification. In addition, there is a possibility for false positive rate with NGS testing, given the fact that certain level, different distribution, or the segmental size of aneuploidy of mosaicism could be harmless. The detection of mosaicism or small-size aneuploidy are still challenged by both the biological and technical biases, which include sampling bias, confined mosaicism, reciprocal errors, artifacts from whole-genome amplification, S-phase artifacts, or smoothing mask from algorithm [47, 48]. Two independent laboratories interpreted the data generated from the same amplified products on the two CCS platforms in this study. Although the interpretation would be made in a double-blind manner, the subjective decisions may be involved in samples without the finest overall backgrounds of CCS. The aCGH is a widely prevalent CCS platform in PGS. However, the accuracy is difficult to be defined merely based on the parallel comparison between aCGH and NGS, since their methodologies are completely different. The array system has been applied and verified in prenatal and postnatal diagnoses for years, other than higher resolution, but also for its efficiency on mosaicism and CNVs micro-deletion and micro-duplication detection. However, the aCGH for preimplantational testings and the arrays for prenatal/postnatal testings involved different probe designs and cell number, and thus with different resolutions. The widely-used aCGH for PGS in this study has broad coverage to the 24 chromosomes, but with comparatively lower resolution. The mosaicism with aneuploid ratio under 40% or segmental aneuploidy with length <5 Mbps are difficult to be detected in clinical application. Therefore, the inconsistency in this study seems to be more likely originated from the methodological limitations of aCGH itself.

Up to now, there is no consensus for the golden standard method in CCS. A future study with the third platform, such as oligo-array or single nucleotide polymorphism (SNP) array could be applied to evaluate the mosaicism and segmental aneuploidy detected with either aCGH or NGS platform. Furthermore, the examinations to samples from miscarriages with PGS-diagnosed mosaic or segmental aneuploid embryo transfer can also clarify the actual effect of detected abnormalities.

## Conclusions

Conclusively, this study demonstrated that the PGS/NGS platform identified the embryos with chromosomal mosaicism and segmental aneuploidy more clearly than that of PGS/aCGH. Upon excluding the transfer of mosaic and segmental-aneuploid embryos, the patients with NGS-screened embryo transfer achieved significantly higher HCG(+) and implantation rates than those with aCGH-screened embryo transfer. The clinical and ongoing pregnancy rates appeared higher, but did not reached statistical significance in the NGS group. A large randomized controlled clinical trial confirming the clinical effectiveness is needed to validate these findings before the extensive use of NGS-based PGS.

## Abbreviations

aCGH: Array comparative genomic hybridization
AFC: Antral follicle count
AMH: Anti-Müllerian hormone
CCS: Comprehensive chromosome screening
CPM: Confined placental mosaicism
ICM: Inner-cell mass
IVF: In vitro fertilization
MII: Metaphase II
NGS: Next-generation sequencing
PGS: Preimplantation genetic screening
SNP: Single nucleotide polymorphism
TE: Trophectoderm
WGA: Whole genome amplification
